# Empowering Expecting Mothers: The Impact of Antenatal Classes on Child Birth Experience

**DOI:** 10.7759/cureus.68299

**Published:** 2024-08-31

**Authors:** Ushna Ahmed, Farheen Yousuf, Zahid H Wadani, Amir Raza

**Affiliations:** 1 Obstetrics and Gynecology, Aga Khan University, Karachi, PAK

**Keywords:** fear of childbirth, childbirth satisfaction, self-efficacy in labor, labor experience, antenatal education

## Abstract

Introduction

Antenatal education is one of the ways to increase a woman’s knowledge about pregnancy and childbirth, which could improve self-efficacy during labor, making the expecting mothers well-prepared for labor and nursing. One of the ways for its delivery could be via well-structured antenatal classes. Such classes are part of many countries’ healthcare systems and have proven to decline maternal anxiety, shorten labor, increase partner involvement, and overall improve labor experience. However, at the same time, such classes can augment more interventions in labor, induction of labor, and epidural usage. Despite the heterogeneous results of their effect, the high demands of antenatal education among pregnant women can justify its incorporation into antenatal care. By allowing the women to identify labor correctly and preventing admission in false labor, shortening the labor, and teaching women non-pharmacologic ways to manage labor pains, it can reduce the patient and fiscal burden on the already overwhelmed maternity units of Pakistan, hence proving to be an inexpensive health promotion tool.

Methods

This cross-sectional study was conducted in the Department of Obstetrics and Gynecology of Aga Khan University Hospital Karachi, Pakistan, between December 2020 and June 2021. All pregnant women, aged 18 to 45 years, between gestational ages of 37 to 42 weeks, with singleton, cephalic pregnancy, booked during the first or second trimester, and who attended at least one antenatal class, were invited to participate. Women who had any contraindication to vaginal delivery, whose labor was induced, or who suffered from medical, psychological, or obstetric comorbidities were excused. The participants were observed for labor outcomes in terms of stage of labor on arrival, use of epidural analgesia, mode of delivery, and childbirth experience, using a validated research tool, known as the Questionnaire for Assessing the Childbirth Experience (QACE) to identify women with a positive or negative childbirth experience. A higher score representing on the questionnaire represented a more negative birth experience. A score of less than 19 was considered a positive birth experience. Data was analyzed using IBM Statistical Package for Social Sciences (SPSS) Statistics for Windows, Version 19.

Results

The mean QACE score was reported as 18.2±3.00, reflecting that on average the mothers had a positive childbirth experience. Modes of delivery revealed 57.6% of the women delivering spontaneously, with 60.4% of them arriving in the labor room in active labor. Demand for labor analgesia in the form of epidural was 64%. Majorly the participants attended only one antenatal class, with 66.91% of women reporting a positive childbirth experience.

Conclusion

Antenatal education classes are a low-input, high-yielding resource that can be used to empower mothers with pertinent information and support for a pleasant childbirth experience while simultaneously taking the edge off the overburdened maternity wards of low resources, in densely populated countries like Pakistan. Hence it is the need of the hour to devise plans to invest in these classes to make them available to the masses.

## Introduction

Childbirth education could improve women’s self-efficacy during labor. Antenatal education is one of the ways to increase a woman’s knowledge about pregnancy and childbirth therefore the expecting mothers could be well prepared for labor and nursing. This could be delivered via well-structured antenatal classes. Antenatal education is a part of the antenatal care system of countries such as Denmark, Brunei, and Australia, among many others, with some even offering pre-pregnancy health education [1,2]. The Danish randomized NEWBORN trial indicated that attending a structured antenatal class increases a woman’s confidence in her ability to deal with labor at home and, hence, results are in favor of stronger childbirth self-efficacy [1-4].

Antenatal education has proven to cause a decline in maternal anxiety, more partner involvement, and less false labor admission [2]. It also shortens the first stage and total duration of labor, with overall better labor experience [3]. A Danish study randomized 435 women to attend a total of three antenatal classes, each three hours in length. It estimated the cervical dilatation on arrival to the maternity ward and the need for analgesia during labor. An estimated 56% of women, exposed to antenatal education, arrive in the maternity ward in the active phase of labor, that is at above 3 cm, as compared to 38% who were not exposed. However, pharmacological analgesia was used by 66% of the exposed, as compared to 71% who were not, which was not statistically significant. Despite that, antenatal classes could be a low-cost, simple, health promotion tool [4].

Nevertheless, despite attending antenatal classes, many women feel unprepared, particularly beyond labor and birth [5]. Some even feel no difference between the amounts of fear experienced with respect to exposure to antenatal classes, declaring no effect of antenatal education classes on childbirth experience [4,6]. The classes may even result in more interventions during labor, induction of labor, and epidural usage during labor [2]. However, the high demand for antenatal education among pregnant women can justify its incorporation into antenatal care [7].

One major concern is that the content of antenatal education has evolved over time but without testing its effect on relevant outcomes [8]. To have an impact as an education tool, antenatal classes should be tailor-made to adapt to each situation and individual’s health requirement, which in turn makes it a highly complex tool to evaluate [7]. The aims of antenatal classes should be to increase the knowledge of women multi-dimensionally, regarding all aspects of childbirth, antenatal, intrapartum, postpartum, as well as psychological aspects of becoming a parent, and beyond [8].

The available literature has speculated that antenatal classes can have a major impact in reducing the fiscal and patient burden of maternity services, favorable for the already overloaded maternity wards of Pakistan - a developing nation struggling to strengthen its health sector to meet public demands. Allowing the women to identify labor correctly can result in less admission of women to the labor room in false labor or in the latent phase of labor. Well-structured antenatal classes can also reduce the need for anesthesia during the latent phase of labor by offering proper non-pharmacological coping mechanisms. Only a few institutes offer antenatal education in Pakistan. Currently, no local literature is available evaluating the impact of these antenatal classes on the birth process. This study aimed to determine the effect of antenatal classes on the labor phase at which women present to the labor ward, and whether they were able to manage the pain without pharmacological intervention, simultaneously determining the impact of such education on the overall self-efficacy experienced by women during labor. We intend to generate local data regarding the impact of antenatal classes on the birth process, premising that attending antenatal classes improves childbirth self-efficacy, creating a more positive childbirth experience for the mothers. Measures can then be designed to incorporate such classes into various maternity setups, as an inexpensive health promotion tool.

## Materials and methods

Study design, study participants, and setting

This was a cross-sectional study, conducted in the Department of Obstetrics and Gynecology at Aga Khan University Hospital, Karachi, Pakistan, over a period of six months. The aim of the study was to assess the outcome in terms of the birth experience, cervical dilatation on arrival to the labor ward, and demand for pharmacological analgesia by the pregnant women arriving for delivery to the labor and delivery suite of the hospital who attended at least one antenatal class. We also aimed to compare the positive childbirth experience with the number of antenatal classes attended by these women using a validated Questionnaire for Assessing the Childbirth Experience (QACE) questionnaire [9]. Pregnant women aged 18 to 45 years, between gestational ages of 37 to 42 weeks, with singleton, cephalic presentation babies, who were booked during their first or second trimesters, and attended at least one antenatal class were invited to participate. Among these women, those who had comorbidities, including maternal medical, obstetrical, or psychological illnesses, or who suffered from prolonged rupture of membranes, fetal growth restriction, history of maternal infertility, oligo or polyhydramnios, or had any contraindication to vaginal delivery or had to undergo induction of labor were excluded.

Sample size and technique

The sample size was achieved using the WHO sample size calculator by taking statistics for three antenatal visits at 36.4%, a margin of error of 8%, and a 95% confidence level. The estimated sample size is 139 [4]. This was a non-probability consecutive sampling technique.

Data collection, measures, and tools

Following approval from the institutional ethics review committee, all laboring women arriving at the labor and delivery suite of Aga Khan University Hospital for delivery and fulfilling inclusion criteria were enrolled for the study after obtaining informed consent. Educational classes are offered to the expecting couple at any time in the prenatal period at the hospital aiming to prepare them for the current pregnancy experience, labor, birth, and early parenthood. A total of four antenatal classes are conducted by the department, one each week. The women are invited at booking, which is usually during the first or second trimester, but they are invited in their second trimester. They teach how to deal with the discomforting symptoms of pregnancy, how to cope with the problems experienced in the second and third trimesters, and to identify true labor correctly, what to do in early labor while at home, and when to go to the hospital, how to cope with the labor experience, and then breastfeeding and care of the newborn, along with self-care during the postpartum period, respectively in these sessions. As the antenatal care appointments in the hospital cover the problems of the second and third trimesters, management of early labor in terms of analgesia at home, labor experience, and immediate post-partum self-care is taught in each class in incremental ways.

All the participants were asked to complete a self-reported survey within 24 hours of birth, which consisted of two modules. The first part assessed their biodata, with demographics and attendance of antenatal classes. The second part explored the childbirth experience, using the QACE, to identify women with positive or negative childbirth experiences. This is a self-reporting questionnaire in French, with a validated English translation available, to assess the childbirth experiences of first-time mothers. It can be used as both a research instrument in its short version and a questionnaire for use in clinical practice in its full version. The short version contained 13 items, with four sub-scales to measure the general assessment of the childbirth experience with scores per dimension. The questions are answered on a 4-point Likert scale, followed by dichotomization of the result to “totally” versus “in part, not so much, not at all.” A score of 1 versus 2 was allocated, respectively. A higher score represents a more negative birth experience. A score of less than 19 was considered as a positive birth experience [9]. Permission was granted to reproduce this assessment tool, provided appropriate credit was given to the original author (developed by Pierre Carquillat, Françoise Vendittelli, Thomas Perneger, and Marie-Julia Guittier), and a link to the creative common license was mentioned (http://creativecommons.org/licenses/by/4.0/).

Participants’ medical records were then used to identify the cervical dilatation at their arrival, and whether any form of pharmacological analgesia was used by them during labor. Cervical dilation of more than 3 cm was labeled as an active phase of labor, indicating greater self-efficacy in dealing with labor at home. Any demand for analgesia in the form of narcotics, nitric oxide gas, or epidural required to control pain was reported under the assumption that women who are more empowered during the birth process will require less pharmacological analgesia during labor.

Recall bias was controlled by time-to-respond constraint (within 24 hours). Confidentiality of the participants was maintained throughout.

Data analysis

Data was entered and analyzed in IBM Statistical Package for Social Sciences (SPSS) Statistics for Windows, Version 19 (IBM Corp., Armonk, USA). Mean and standard deviation were calculated for maternal age, height, weight, body mass index (BMI), gestational age at presentation to labor ward, cervical dilatation on arrival, QACE score, and number of antenatal visits. Frequency and percentages were calculated for educational level, type of labor, demand for labor analgesia, childbirth experience, and mode of delivery. A comparison of positive childbirth experiences and a number of antenatal classes was done using the Chi-square test or Fisher's exact test. Effect modifiers, like maternal age, BMI, gestational age, education levels, type of labor, and mode of delivery, were assessed through stratification. Post-stratification, the Chi-square test was applied. Multivariate logistic regression was used to observe the factors associated with positive birth experience and adjusted odds ratio, with a 95% CI reported. A P-value of less than or equal to 0.05 was considered a significant association.

## Results

The demographics of the participants are displayed in Table 1. Of the 139 patients, ages ranged from 24 to 40 years, with the majority of the participants (62.6%) being graduates of universities. The mean QACE score was reported as 18.2±3.00, indicating on average the mothers had a positive childbirth experience. Regarding the mode of deliveries, a major pool of the participants (57.6%) delivered spontaneously, which could be attributed to a better part arriving at the labor room in active labor (60.4%). However, there was a preponderance of demand for labor analgesia, as 64% of the mothers requested epidural analgesia.

**Table 1:** Demographic and gynecological characteristics (n=139) SVD, spontaneous vaginal delivery; IVD, instrumental vaginal delivery; ECS, emergency cesarean section

Variables	Statistics
Age (years)	31.04±3.96
Gestational age (weeks)	38.69±0.83
BMI (kg/m^2^)	27.4±3.48
Height (cm)	162±2.85
Weight (kg)	72.5±9.74
Education	
Intermediate	26 (18.7%)
Graduate	87 (62.6%)
Postgraduate	26 (18.7%)
Mode of labor	
SVD	80 (57.6%)
IVD	24 (17.3%)
ECS	35 (25.2%)
Demand for labor analgesia	
Yes	89 (64%)
No	50 (36%)
Cervical dilation	
Active phase	84 (60.4%)
Latent phase	55 (39.6%)
Number of antenatal classes	
1	83 (59.7%)
2	51 (36.7%)
3	5 (3.6%)

Majorly the participants attended only one antenatal class and Table 2 demonstrates the comparison of said demographics with respect to the number of classes attended. As none of the participants attended all four classes, we grouped the participants into two groups: the ones who attended a single class and those who attended two or three classes, i.e., multiple classes. The attendees of single versus multiple classes were similar with respect to demographics and education levels, as well as labor and delivery characteristics.

**Table 2:** Comparison of characteristics of women who attended single and multiple classes for childbirth experience SVD, spontaneous vaginal delivery; IVD, instrumental vaginal delivery; ECS, emergency cesarean section

Variables	Single class attended, n=83	2-3 classes attended, n=56	P-value
Age groups			0.464
18-30	54 (65.1%)	33 (58.9%)	
31-45	29 (34.9%)	23 (41.1%)	
BMI (kg/m^2^)			0.735
Non-obese	60 (72.3%)	39 (69.6%)	
Obese	23 (27.7%)	17 (30.4%)	
Education status			0.794
Intermediate	14 (16.9%)	12 (21.4%)	
Graduate	53 (63.9%)	34 (60.7%)	
Postgraduate	16 (19.3%)	10 (17.9%)	
Mode of labor			0.724
SVD	46 (55.4%)	34 (60.7%)	
IVD	16 (19.3%)	8 (14.3%)	
ECS	21 (25.3%)	14 (25%)	
Demand for labor analgesia			0.257
Yes	50 (60.2%)	39 (69.6%)	
No	33 (39.8%)	17 (30.4%)	
Cervical dilatation on arrival to labor			0.209
Active phase	46 (55.4%)	37 (66.1%)	
Latent phase	37 (44.6%)	19 (33.9%)	

**Figure 1:**
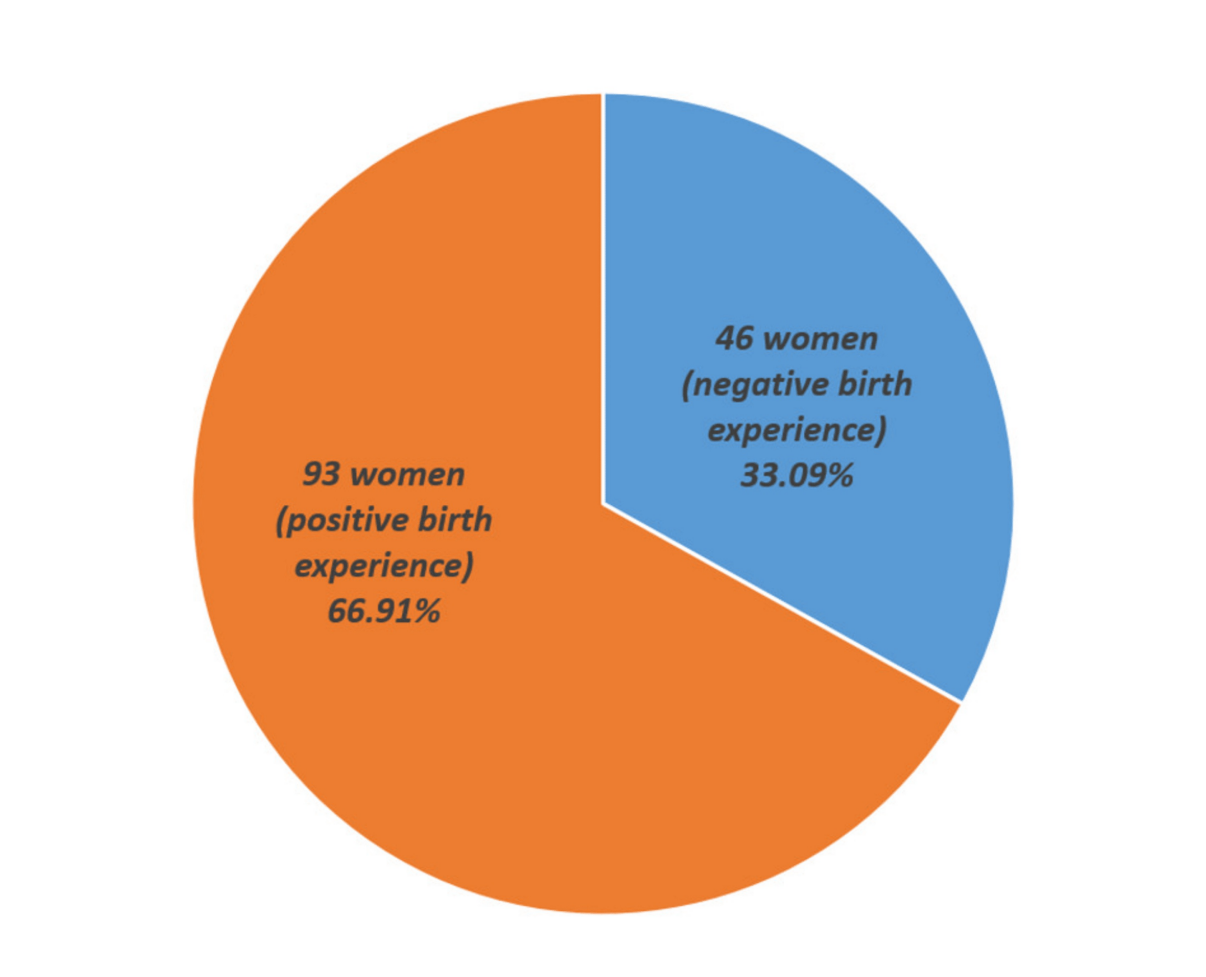
Birth experience of women after antenatal classes (n=139)

Within the participants, the overall childbirth experience was pleasant, with 66.91% of the participants having a positive experience against 33.09% experiencing a more negative day, as expressed in Figure 1.

Table 3 compares the characteristics of the participants against their childbirth experiences. Women who experienced a positive or negative childbirth were found to fall in the similar age bracket of 18-30 years, expressed the same educational status, and were non-obese. A better part of both groups delivered spontaneously vaginally, arriving in the labor ward in the active labor phase, but demanded labor analgesia.

**Table 3:** Comparison of characteristics of women with positive and negative birth experiences SVD, spontaneous vaginal delivery; IVD, instrumental vaginal delivery; ECS, emergency cesarean section

Variables	Positive birth experience, n=93	Negative birth experience, n=46	P-value
Age groups			0.653
18-30	57 (61.3%)	30 (65.2%)	
31-45	36 (38.7%)	16 (34.8%)	
BMI (kg/m^2^)			0.761
Non-obese	67 (72%)	32 (69.6%)	
Obese	26 (28%)	14 (30.4%)	
Education status			0.244
Intermediate	21 (22.6%)	5 (10.9%)	
Graduate	55 (59.1%)	32 (69.6%)	
Postgraduate	17 (18.3%)	9 (19.6%)	
Mode of labor			0.167
SVD	56 (60.2%)	24 (52.2%)	
IVD	18 (19.4%)	6 (13%)	
ECS	19 (20.4%)	16 (34.8%)	
Demand for labor analgesia			0.585
Yes	61 (65.6%)	28 (60.9%)	
No	32 (34.4%)	18 (39.1%)	
Cervical dilatation on arrival to labor			0.845
Active phase	55 (59.1%)	28 (60.9%)	
Latent phase	38 (40.9%)	18 (39.1%)	

**Figure 2:**
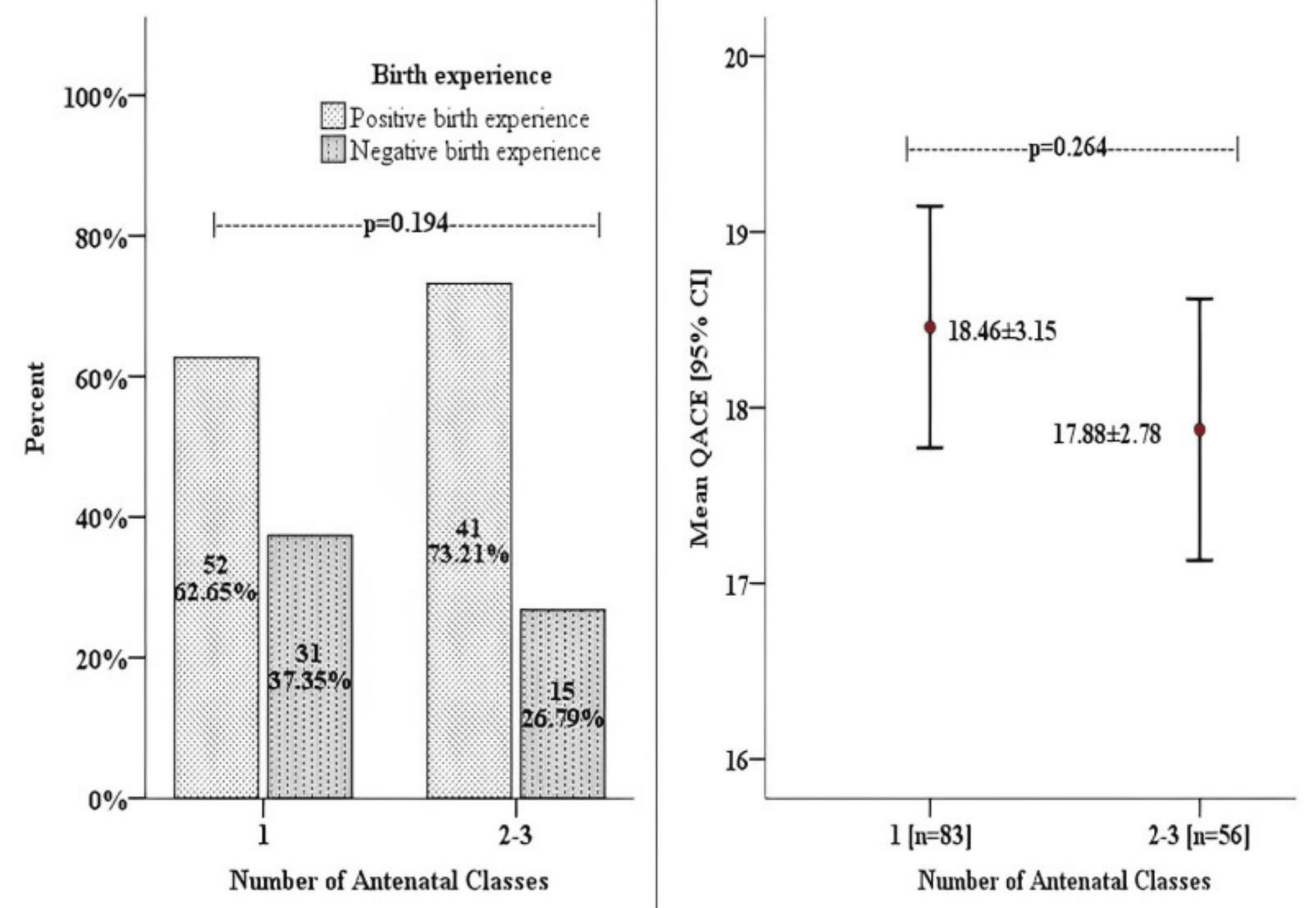
Comparison of birth experience in terms of proportion and mean QACE between women who attended single and multiple classes QACE, Questionnaire for Assessing the Childbirth Experience

Of the participants who attended a single antenatal class, 62.65% of them had a positive childbirth experience and of those who attended two to three classes, 73.21% had a positive childbirth experience. Women who attended more than one class had a lower QACE score than those who attended a single antenatal class (17.88±2.78 vs. 18.46±3.15) (Figure 2).

Among the women who attended at least one antenatal class, the majority reported a positive childbirth experience (66.91%). The participants who experienced positive and negative childbirth experiences belonged to similar demographics in terms of age group and education level and were mostly non-obese. There was no statistical difference between the two groups with regards to obstetric characteristics of mode of delivery and arrival to the labor ward in active labor, and the bulk of both groups demanded epidural analgesia. Despite sharing similar demographics, women who experienced a positive childbirth attended a greater number of classes. In fact, women who attended two to three classes scored lower on the QACE score, which indicated a greater self-efficacy during labor [9].

In the present study, women who felt more in control of their labor and less worried had greater partner involvement, attended a greater number of antenatal classes, arrived at the labor ward in active labor but demanded epidural analgesia, with similar age brackets, educational status, and BMI as women who scored more toward a negative experience, depicted by Table 4.

**Table 4:** Multivariate analysis showing the factors associated with positive birth experience Ref, reference; SVD, spontaneous vaginal delivery; IVD, instrumental vaginal delivery; ECS, emergency cesarean section

Factors	Adjusted odds ratio	95% CI of odds ratio	P-value
Antenatal classes			0.23
1	Ref		
2-3	1.6	0.74-3.47	
Age groups			0.602
18-30	Ref		
31-45	1.24	0.56-2.75	
BMI (kg/m^2^)			0.853
Non-obese	Ref		
Obese	0.92	0.41-2.08	
Education status			
Intermediate	Ref		
Graduate	0.44	0.18-1.32	0.14
Postgraduate	0.46	0.12-1.72	0.28
Mode of labor			
SVD	Ref		
IVD	1.53	0.52-4.49	0.44
ECS	0.54	0.23-1.26	0.15
Demand of labor analgesia			0.86
Yes	1.07	0.49-2.34	
No	Ref		
Cervical dilatation on arrival to labor			
Active phase	Ref		
Latent phase	1.04	0.48-2.27	0.91

## Discussion

The demographics and obstetric characteristics of women with a more pleasant and a more unpleasant childbirth experience were similar. Those who attended a single antenatal class shared the same demographics and obstetrics characteristics as those who attended more than one class. The majority of the participants had spontaneous vaginal deliveries, which can be attributed to their arrival to the labor ward in the active phase but demanded epidural analgesia despite feeling more in control of their labor. Most of the mothers experienced pleasant childbirth, with a lower QACE score among those who attended more than one class, indicating higher self-efficacy experienced intrapartum. Childbirth self-efficacy reflects a woman’s trust in her ability to cope with labor and birth. It influences a woman's perception of her childbirth and can determine it to be happy or sad [9,10]. Antenatal classes have unanimously proved to enhance childbirth self-efficacy and ability to handle the birth process, with fewer women feeling low confidence in their ability to cope at home during labor [1,6,10,11]. Self-efficacy can be affected by one’s previous experiences, but in the absence of it, one is highly influenced by the persuasion of others [11,12]. Antenatal classes can bridge this gap between childbirth self-efficacy and lack of experience, giving mothers a sincere sense of control over their labor. Different validated scales and scores have all concluded less anxiety and more comfort and confidence in women regarding handling their labor, especially exceptional decision-making ability, after gaining antenatal education [2,8,9,11-13].

The demographics and obstetric characteristics of women experiencing contrasting childbirth within parallel antenatal education exposure were observed in a few studies distinctly evaluating them, with the conclusion that neither age nor education status has any impact on childbirth experience [3,12]. The mothers sharing similar age groups, gestational age, and BMI regarded other's experience of labor and delivery and even firmly their own previous experience of pregnancy and delivery greatly impacting their experience of current childbirth in terms of anxiety and self-assurance, an impact which could not be studied in our pool of exclusively primigravida women [12-14]. This impact has been found to be counterproductive at times, as it can lead to increased fear of labor or tocophobia, which is the most common cause of cesarean section [7,15]. Evidence is contradictory at best for antenatal education impacting modes of delivery, with observational studies reporting antenatal classes not affecting the mode of delivery, inconclusive results declared in large systemic analyses from different continents, and a qualitative study even declaring a reduction in cesarean section rate among attendees of said classes [2,3,8,16,14].

It has been hoped and aimed to use these antenatal classes as a means to inculcate knowledge for the management of labor pains without pharmacological agents while simultaneously educating about the pharmacological options. This, however, has ricocheted, as the majority of the studies indicate their participants demanding epidural analgesia, more so in the latent phase of labor, which was also reflected in our study [2,3,8]. A few small studies suggest similar or sometimes a reduced need for epidural analgesia by the attendees, but the data is heterogeneous and inconclusive [12,15,17,18]. Despite the augmented use of epidural analgesia, an extensive qualitative study suggests that women want to know more about the options to manage labor pains, be it pharmacological or otherwise, and look toward classes as official means for it, as every woman's experience with analgesics varies and does not provide assistance to new mothers in choosing a method best suited for them by the word of mouth [14]. This persuades argument in the favor of continuing to educate women about epidural and other labor analgesic methods in antenatal classes.

One of the core objectives of antenatal classes has always been to alleviate patient burden on the saddled maternity wards by allowing women to manage a part of labor at home so that they present to the labor ward in a more advanced stage. Our study does reflect this as the majority of the participants presented in the active phase of labor. This is the most supported variable in several observational and interventional studies conducted throughout, with women who took part in antenatal classes arriving in active labor and strongly desiring antenatal classes to deliver them information to identify labor correctly to avoid going back and forth from the labor ward [2,3,4,8,12,14,16,17].

Our study indicated a lower QACE score among the majority of the participants attending the antenatal classes, which indicates greater self-efficacy in labor. In the absence of past experience, self-efficacy can be highly influenced by the persuasion of others, shifting the argument in favor of antenatal classes to tame this [19]. There is a dearth of indigenous data arising from the developing world, and none from Pakistan, regarding the impact of antenatal classes on the birth process and outcome. In fact, certain systematic reviews even excluded the few available studies from the developing world owing to the poor organization of the health care system here [4,5]. Our study was able to generate local literature reflecting the effectiveness of antenatal classes, as they are offered in only scanty maternity units of Pakistan that too only in tertiary care centers of metropolitan cities. This local data can lead to persuading governing authorities to advocate and invest in these low-input, high-output yielding education tools. Our study indicated a lower QACE score among the majority of the participants attending the antenatal classes, which indicates greater self-efficacy in labor.

Antenatal education now often offers some preparation of labor and birth for the father, thus enhancing partner involvement in labor. It also offers breastfeeding information and strategies to cope with the postbirth adjustment: social, physical, and psychological, all welcoming and highly demanded knowledge [8,14,20,21]. It is increasingly seen that the perception of greater self-confidence in labor is not affected by the feeling of pain but could be attributed to greater partner involvement and greater knowledge of coping mechanisms and what to expect in labor and after delivery with regards to caring for the infant [13,14,20,21]. All this information is either already included or could be added to the syllabus of these classes.

Despite this ambition, the study has its limitations. By including only first-time mothers, it cannot evaluate how previous labor experiences would have affected the participants’ recent experience, now equipped with the new knowledge of labor and delivery beforehand. It excluded a chunk of pregnant women with comorbidities who could have benefitted from such classes. It is conducted in one tertiary care private hospital of a developing nation, and results cannot be generalized for the other cities or even other public sector maternity setups of the country. Nevertheless, this is in an effort to stimulate maternity centers around the country to begin investing in antenatal education.

## Conclusions

Antenatal classes require limited resources in terms of funds, infrastructure, manpower, and space. They can be conducted in the same premises where other antenatal care is provided, yet they continue to provide invaluable benefits in terms of greater self-efficacy and less anxiety during labor, hence, a happier and more satisfying childbirth experience. Regardless of inconclusive and heterogeneous conclusions of their impact in the index and various other studies, these classes are highly demanded for information provision and reassurance, meeting women in similar circumstances for support and fraternity, ease in transition to parenthood for both parents, preparing effective support during labor, and simultaneously can relieve some burden over the maternity wards by allowing women to identify labor correctly and present in an advanced stage. It is the need of the hour for investment in such a low-input resource that empowers and equips mothers with the pertinent information and skills to ensure a positive childbirth experience. This is particularly important for low-resource, densely populated countries to support their maternity services so they can provide equity of care.

## Figures and Tables

**Figure 3:**
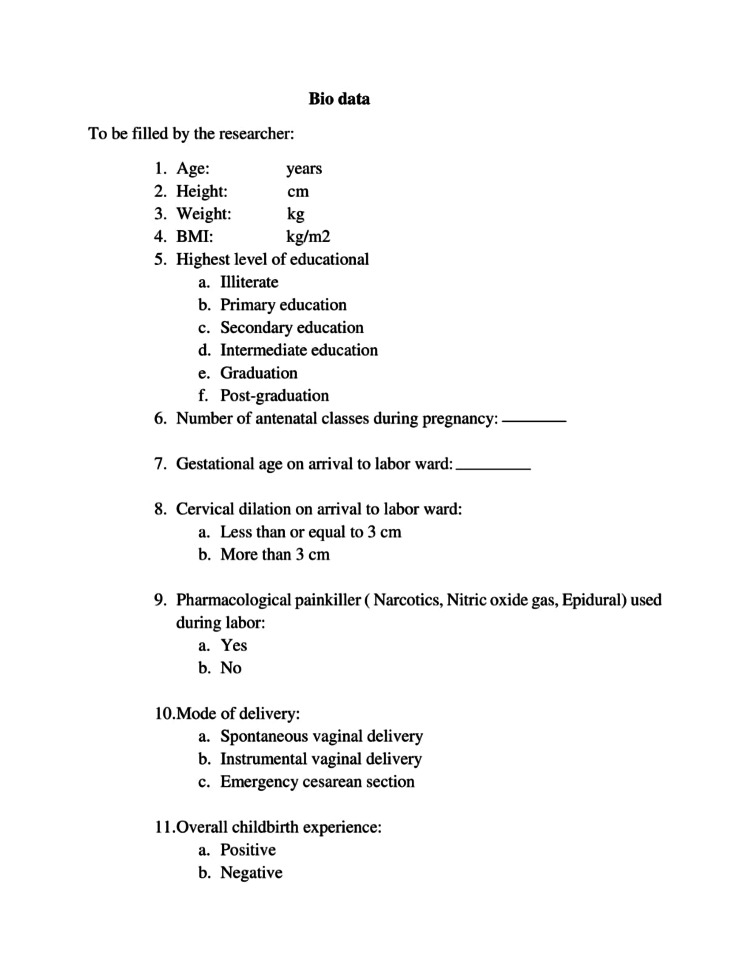
Biodata to be filled using electronic medical records

**Figure 4:**
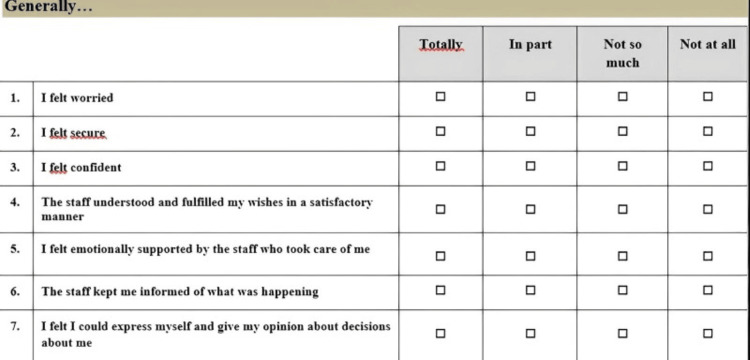
Generally Permission was granted by the original publishers to reproduce the questionnaire.

**Figure 5:**
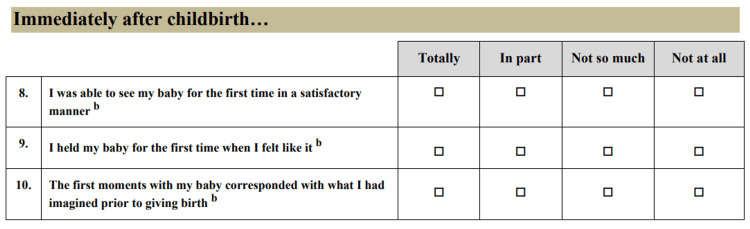
Immediately after childbirth a: Dichotomization "not at all" versus "not so much, in part, totally" ( score of 1 versus 2 respective); b: Dichotomization "totally" versus "in part, not so much, not at all" (score of 1 versus 2 respective). Permission was granted by the original publishers to reproduce the questionnaire.

**Figure 6:**
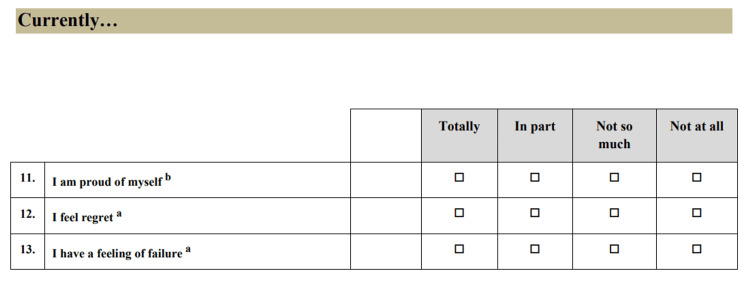
Currently a: Dichotomization "not at all" versus "not so much, in part, totally" (score of 1 versus 2 respective); b: Dichotomization "totally" versus "in part, not so much, not at all" (score of 1 versus 2 respective). Permission was granted by the original publishers to reproduce the questionnaire.

## Appendices

Questionnaire

Biodata is sown in Figure 3.

The Questionnaire for Assessing the Childbirth Experience (QACE), vaginal birth or cesarean [9]. The first part assesses the feelings of the mother regarding childbirth in general (Figure 4).

The second part assesses the feelings of the mother immediately after childbirth (Figure 5) [9].

 The third part assesses the feelings of the mother at the current moment (Figure 6) (9).

## References

[1] Brixval CS, Axelsen SF, Thygesen LC, Due P, Koushede V (2016). Antenatal education in small classes may increase childbirth self-efficacy: results from a Danish randomised trial. Sex Reprod Healthc.

[2] Ferguson S, Davis D, Browne J (2013). Does antenatal education affect labour and birth? A structured review of the literature. Women Birth.

[3] Yohai D, Alharar D, Cohen R (2018). The effect of attending a prenatal childbirth preparedness course on labor duration and outcomes. J Perinat Med.

[4] Maimburg RD, Vaeth M, Dürr J, Hvidman L, Olsen J (2010). Randomised trial of structured antenatal training sessions to improve the birth process. BJOG.

[5] Buultjens M, Murphy G, Robinson P, Milgrom J, Monfries M (2017). Women's experiences of, and attitudes to, maternity education across the perinatal period in Victoria, Australia: a mixed-methods approach. Women Birth.

[6] Jafari E, Mohebbi P, Mazloomzadeh S (2017). Factors related to women’s childbirth satisfaction in physiologic and routine childbirth groups. Iran J Nurs Midwifery Res.

[7] Artieta-Pinedo I, Paz-Pascual C, Grandes G, Remiro-Fernandezdegamboa G, Odriozola-Hermosilla I, Bacigalupe A, Payo J (2010). The benefits of antenatal education for the childbirth process in Spain. Nurs Res.

[8] Brixval CS, Axelsen SF, Lauemøller SG, Andersen SK, Due P, Koushede V (2015). The effect of antenatal education in small classes on obstetric and psycho-social outcomes - a systematic review. Syst Rev.

[9] Carquillat P, Vendittelli F, Perneger T, Guittier MJ (2017). Development of a questionnaire for assessing the childbirth experience (QACE). BMC Pregnancy Childbirth.

[10] Çankaya S, Şimşek B (2021). Effects of antenatal education on fear of birth, depression, anxiety, childbirth self-efficacy, and mode of delivery in primiparous pregnant women: a prospective randomized controlled study. Clin Nurs Res.

[11] Duncan LG, Cohn MA, Chao MT, Cook JG, Riccobono J, Bardacke N (2017). Benefits of preparing for childbirth with mindfulness training: a randomized controlled trial with active comparison. BMC Pregnancy Childbirth.

[12] Ricchi A, La Corte S, Molinazzi MT, Messina MP, Banchelli F, Neri I (2020). Study of childbirth education classes and evaluation of their effectiveness. Clin Ter.

[13] AlSomali Z, Bajamal E, Esheaba O (2023). The effect of structured antenatal education on childbirth self-efficacy. Cureus.

[14] Spiby H, Stewart J, Watts K, Hughes AJ, Slade P (2022). The importance of face to face, group antenatal education classes for first time mothers: A qualitative study. Midwifery.

[15] Kacperczyk-Bartnik J, Bartnik P, Symonides A, Sroka-Ostrowska N, Dobrowolska-Redo A, Romejko-Wolniewicz E (2019). Association between antenatal classes attendance and perceived fear and pain during labour. Taiwan J Obstet Gynecol.

[16] Hong K, Hwang H, Han H (2021). Perspectives on antenatal education associated with pregnancy outcomes: systematic review and meta-analysis. Women Birth.

[17] Cutajar L, Miu M, Fleet J-A, Cyna AM, Steen M (2020). Antenatal education for childbirth: labour and birth. Eur J Midwifery.

[18] Cutajar L, Cyna AM (2018). Antenatal education for childbirth-epidural analgesia. Midwifery.

[19] Uludağ E, Serçekuş P, Vardar O, Özkan S, Alataş SE (2022). Effects of online antenatal education on worries about labour, fear of childbirth, preparedness for labour and fear of covid-19 during the covid-19 pandemic: a single-blind randomised controlled study. Midwifery.

[20] Suto M, Takehara K, Yamane Y, Ota E (2017). Effects of prenatal childbirth education for partners of pregnant women on paternal postnatal mental health and couple relationship: a systematic review. J Affect Disord.

[21] Serçekuş P, Başkale H (2016). Effects of antenatal education on fear of childbirth, maternal self-efficacy and parental attachment. Midwifery.

